# A fuzzy model for processing and monitoring vital signs in ICU patients

**DOI:** 10.1186/1475-925X-10-68

**Published:** 2011-08-03

**Authors:** Cicília RM Leite, Gláucia RA Sizilio, Adrião DD Neto, Ricardo AM Valentim, Ana MG Guerreiro

**Affiliations:** 1 Department of Informatic - State University of Rio Grande do Norte/College of Science and Technology Mater Christi - Brazil, Av. Campus Universitário - BR 110, KM, 46 - CEP: 59625-620 - Brazil; 2 Department of Biomedical Engineering, Federal University of Rio Grande do Norte (UFRN), Caixa Postal 1524 - Campus Universitário - UFRN/CT/DCA - CEP: 59072-970 - Brazil

## Abstract

**Background:**

The area of the hospital automation has been the subject of much research, addressing relevant issues which can be automated, such as: management and control (electronic medical records, scheduling appointments, hospitalization, among others); communication (tracking patients, staff and materials), development of medical, hospital and laboratory equipment; monitoring (patients, staff and materials); and aid to medical diagnosis (according to each speciality).

**Methods:**

In this context, this paper presents a Fuzzy model for helping medical diagnosis of Intensive Care Unit (ICU) patients and their vital signs monitored through a multiparameter heart screen. Intelligent systems techniques were used in the data acquisition and processing (sorting, transforming, among others) it into useful information, conducting pre-diagnosis and providing, when necessary, alert signs to the medical staff.

**Conclusions:**

The use of fuzzy logic turned to the medical area can be very useful if seen as a tool to assist specialists in this area. This paper presented a fuzzy model able to monitor and classify the condition of the vital signs of hospitalized patients, sending alerts according to the pre-diagnosis done helping the medical diagnosis.

## Background

Due to the large volume of information involved and operations to be performed in hospital automation processes, the control and data management that support information to base decision making become quite complex.

In this environment, permeated by the deployment of new technologies, which involve modeling and/or simulations of real environments and medical applications development aimed at optimizing the processes of health care, research in this area contribute to improving the quality of services as an instrument that allows to optimize care and minimize risk to patient health. These factors contribute to improving the quality of population health.

The use of Intelligent Systems (IS) techniques [1] in patient monitoring ([2-9] and [10]) for helping in medical diagnosis ([11-16] and [17]) has been established as an expanding area of research using its features and capabilities to turn data into useful information. Thus, several studies were identified whose main theme is devoted to the process of monitoring the patients. Next will be briefly described some references that had major impact in this research. A data mining system for monitoring chronic patients by examining the QRS complex signal coming from the electrocardiogram (ECG) has been developed in Tseng [2]. Already Murakami [3] developed the vMonGluco, which implements the real-time monitoring of patients glucose levels, developed for mobile devices and showing that tight control of blood glucose levels is beneficial for diabetic patients. Varshney [4] presented some specific requirements for the conduct of patient monitoring, proposing wireless networks model-oriented in the monitoring process. Leite [5] used stochastic Petri Nets in modeling and simulation of medical care performed in the ICU patients. Varady [6] presented an open architecture system for patient monitoring and Spode [7] developed a non-invasive remote monitoring system of vital signs. Baura [8] showed that monitoring patients is a process that requires continuous observation, characteristic that requires availability guarantees of the monitoring systems. This argument has been strengthened by Van den Berghe [9] showing that restrictive monitoring blood glucose levels can reduce mortality among critically patients in an ICU. In this way, Shin [10] also developed a research oriented to patients monitoring, applying fuzzy to pre-diagnosis inference.

Several papers focused on helping medical diagnosis were also observed. Soares [11] presented a new intelligent methodology for analysis and classification of skin cancer images, based on the techniques of digital image processing for color, shape and texture feature extraction, using the Wavelet Packet Transform (WPT) and learning techniques of Support Vector Machine (SVM - Support Vector Machine). Rogal Jr [12] used an Artificial Neural Network (ANN) ART2 in the group of cardiac arrhythmias, rating normal heartbeat, atrial premature contractions (APC) and ventricular premature contractions (CPV). Jara [13] proposed an intelligent information system to detect and predict myocardial diseases using medical data for vital signs, first, to detect symptoms through a system of rules and, moreover, make the disease prediction through chronobiology algorithms. At the same time, Koutsojannis [14] developed the HIROFILOS-II, a hybrid intelligent system for diagnosis and treatment of prostate diseases based on symptoms and test results of patients' health records. The main part of HIROFILOS-II is built by the rule extraction of patient records using machine learning techniques, and then, manually, turning them into fuzzy rules.

Leite [15] undertook the classification of cardiac arrhythmias through electrocardiogram descriptors (ECG) using Kohonen competitive neural networks, detecting whether the ECG showed any cardiac arrhythmia. Zhu conducted a research [16] related to the automatic detection of glucose in blood abnormalities using a machine learning approach. Barakat [17] introduced the use of Support Vector Machines (SVM) for diagnosis of Diabetes Mellitus.

Considering the literature reviewed, it is noted that patients monitoring should be an automation process, addressing the transmission of patient's vital signs through the hospital network.

Considering the literature reviewed, it is noted that patients monitoring follows the line of automation of the monitoring process, approaching the transmission of patient's vital signs for the hospital network, not worrying about these signals processing (in order to subsidize a diagnosis of monitored patients). From the perspective of diagnostic help, the examined research look for the development of expert systems applied to help medical diagnosis, using artificial intelligence techniques in conducting specific pre diagnosis and looking for, by the application of intelligent techniques, the completion of data processing, transforming them into useful information aimed to assist the diagnosis.

Given the context under study, in this paper is presented an architecture able to transform the multiparameter monitor data into useful information, through the knowledge of specialists and normal parameters of vital signs based on fuzzy logic that allows to extract information about the clinical condition of ICU patients and give a pre-diagnosis. It is still possible that the professionals carry out the patients' monitoring remotely where the medical staff can perform a pre-diagnosis without even being in front of the patient.

### Hospital Automation

The automation can be considered a multidisciplinary area involving: programming languages (software), electronic platforms (hardware) and actuators (mechanical). This factor means that studies in automation are comprehensive and therefore involve a wide range of knowledge [18].

According to Feng [19], hospital automation is a subfield of automation that seeks to promote the automation of processes relevant to the hospital environment, looking for efficiency and productivity and considering mainly the features and constraints peculiar to the medical environment (eg, the acquisition of data should be provided with privacy in order to ensure the ethics of the medical act and preserve the integrity of the patient).

Typically, the hospitals make use of technologies that provide greater security, reliability, robustness to the daily tasks, mainly because they deal with human lives. As an example, we can mention applications (hardware/software), relating to:

**• management and control: **electronic medical records, appointment scheduling, control of pharmacy, hospitalization, laboratory, among others;

**• communication: **tracking patients, staff and materials;

**• medical equipment and laboratory devices:**cardiac monitors, pulse oximeter, stethoscopes, thermometers, surgical tools, magnetic resonance, scanner, among others;

**• monitoring: **patients, staff and materials

**• helping medical diagnosis: **according to each specialty.

Integrating the hospital environment are the ICUs, defined as hospital units for the care of critically or at risk patients who have nonstop medical and nursing assistance, with their own specific equipment and specialized human resources with access to other technologies for diagnosis and therapy [20].

The scope of work includes the monitoring of vital signs of patients in the ICU, aiming at the realization of pre-diagnostic to help medical decision and providing information to send alerts when needed.

### Patient and Vital Signs

According to Hoerr [21]: "the patient is any individual under medical care." Particularly, patients in the ICU need care, company and continuous monitoring of vital signs, aimed at early detection of risk situations, allowing timely intervention by health professionals monitoring of vital signs, to early detect risk situations, allowing timely intervention by medical staff. Some patients are admitted because they present a severe clinical manifestation and others are admitted only for monitoring of vital signs because they underwent surgery, for example. So, the automation process of the ICU becomes necessary in order to minimize errors and maximize available resources to the medical team for the patients' care and monitoring.

Vital signs are measurements that provide physiological data indicating the health conditions of the person, demonstrating the functioning and changes in body function [22]. Thus, the vital signs guide the initial diagnosis and the monitoring of patients' clinical evolution. So, their main objective is to help in the health assessment of the person, as well as equip the decision making process related to specific interventions. The vital signs monitored that help the medical diagnosis are: systolic blood pressure, diastolic blood pressure, mean arterial pressure, heart rate, respiratory rate, body temperature and partial oxygen saturation, as shown in Table 1[23]. The correct measurement of vital signs is extremely important for the clinical assessment of patients that are, in this work scope, in the ICU.

**Table 1:** Key parameters for patients' vital signs analysis Sample table title [23]

Name	Abbreviation	Normal Value
Pressure (Systolic/Diastolic) *	Bp	04 years old -85/60 mmHg; 06 years old-95/62 mmHg; 10 years olsd - 100/65 mmHg; 12 years old - 108/67 mmHg; 16 years old - 118/75 mmHg; Adults - 120/80 mmHg; Elderly - 140 a 160/90 to 100 mmHg.
**Heart rate** **(The objective is to evaluate whether the heart is beating, and if he does so with appropriate pace and frequency.)**	Hr	Newborn - 100 a 160 bpm Children - 80 a 120 bpm Adult - 60 to 100 bpm
**Respiratory Rate** **(Through rhythm, sound and depth, it reflects the metabolic state of the body, the condition of the diaphragm and chest muscles, supplying oxygen (O2) to the respiratory tract and alveoli.)**	Rr	Newborn- 30 to 60 mrpm Children- 20 to 30 mrpm Adult- 12 to 20 mrpm
**Body Temperature** **(It represents the balance between heat production and heat loss.)**	Bt	Axillary: 36°C to 37°C Oral: 36,2°C to 37,2°C Rectal: 36,4°C to 37,4°C
**Partial oxygen saturation** **(It represents the partial oxygen saturation in the blood.)**	POS_2_	Low- 0 - 94% Normal- 95 - 100%

*MBP - Mean Blood Pressure.

## Methods

As an alternative to monitoring vital signs and conducting pre-diagnosis, object of this work, we consider that the systems for medical decision support using intelligent systems techniques combined with technologies that integrate mobility and portability in accessing processed information. The effects of this architecture can be significant, allowing a better interface, especially in the aspect of expert knowledge, communication and usability, important features for applications in medicine.

**Figure 1:**
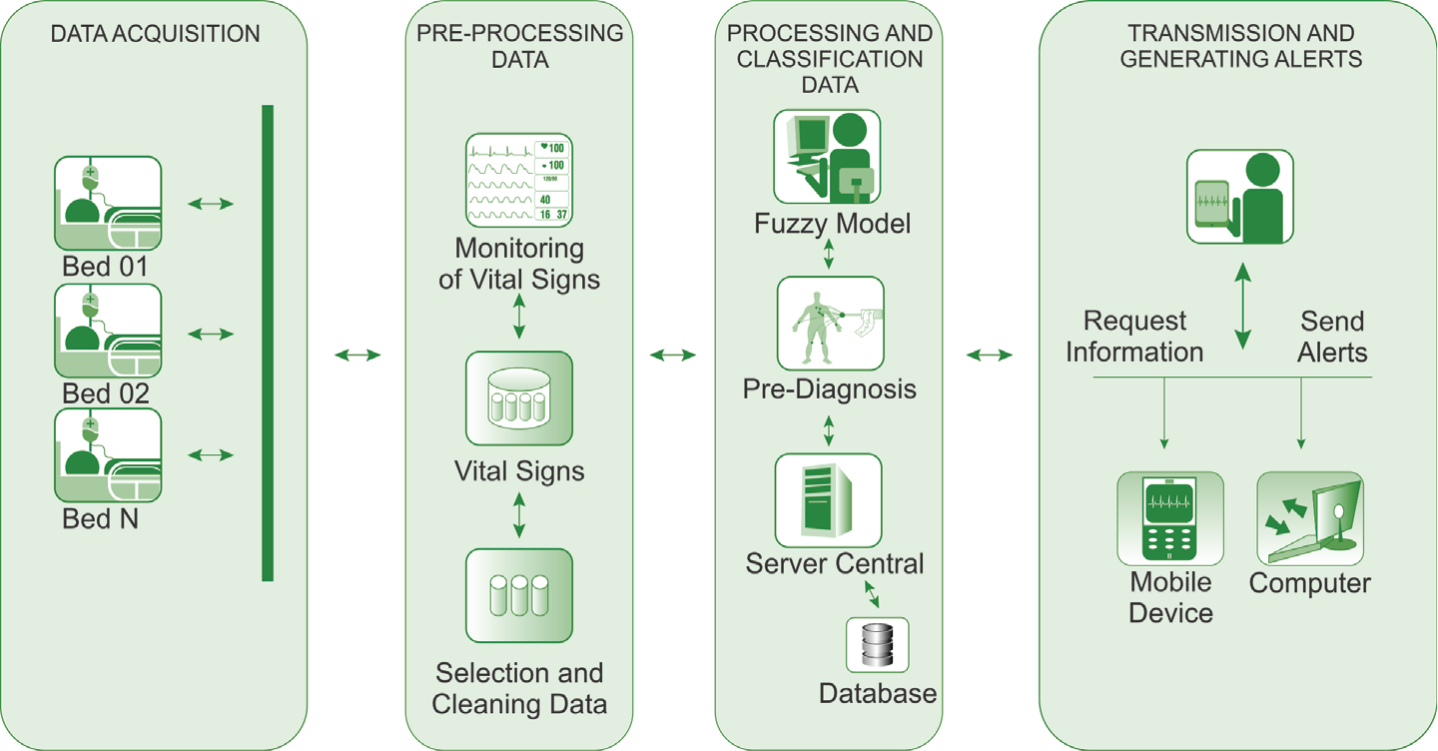
**Architecture of intelligent system for monitoring patients**.

Thus, the specification of the application architecture considered environments with heterogeneous architectures and was based on: the acquisition of data from patient's vital signs monitoring; the use of intelligent systems techniques, especially fuzzy logic; information processing; and sending alerts through mobile devices. The monitored environment, entitled Intelligent System for Monitoring Patients (ISMp) (Figure 1), consists of: acquisition of data through a network of sensors placed in the patients' beds; pre-processing, where the preparation (filtering) and data selection are carried out; data processing and classification, where process is done through fuzzy logic in order to implement a pre-diagnosis to help the medical staff; data post-processing and preparation for sending alerts if any abnormality was detected; the information is sent to mobile devices that are registered in the environment, to support medical staff in decision making and implementation of relevant actions.

### Data Acquisition

To simulate the physiological data acquisition of patients (vital signs), we used the MIMIC (Multi-parameter Intelligent Monitoring for Intensive Care), which is a public database available on the Internet by the Physionet [24], in order to assist the work development facing the automation of hospital systems related to multiparametric monitoring of patients. We used the software MATLAB (MathWorks) to read and load the data acquired from the biomedical devices.

The MIMIC has 74 records, with 20 to 40 hours of continuously recorded data each, related to patients admitted for medical, surgical and/or cardiac treatment in the ICU of Beth Israel Hospital in Boston. The data was obtained directly from the heart monitors installed in the beds and each record typically consists of hundreds of individual files.

**Figure 2:**
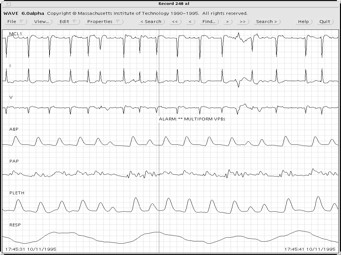
**A MIMIC sample (record 248)**.

It was observed that there are other notes in most of the records in the MIMIC database, including the QRS complex (which compose the ECG signal), as well as periodic alarms related to changes in the patient's condition, including heart and respiratory rate, oxygen saturation and systolic, diastolic and mean pressure shown in Figure 2. In some records data from temperature sensors and cardiac output were also presented.

### Pre-Processing Data

We carried out the extraction of the major physiologic signals that interfere directly in the clinical condition of patients with a stroke diagnosis (mean blood pressure, systolic blood pressure, diastolic blood pressure and oxygen saturation). It was observed that there are other physiological signals in the records of MIMIC database, such as the QRS complex (which compose the ECG signal) periodic alarms related to changes in the patient's condition; heart and respiratory rates; temperature; and cardiac output. The process of acquisition and validation of knowledge was done through weekly interviews during 2 (two) years, at the Promater Hospital, with the ICU medical staff. Thus, by pre-analysis performed together with medical specialists (general practitioner, cardiologist and neurologist) and nurses, the patients for validating the fuzzy model were selected.

### Processing and Classification Data

**Figure 3:**
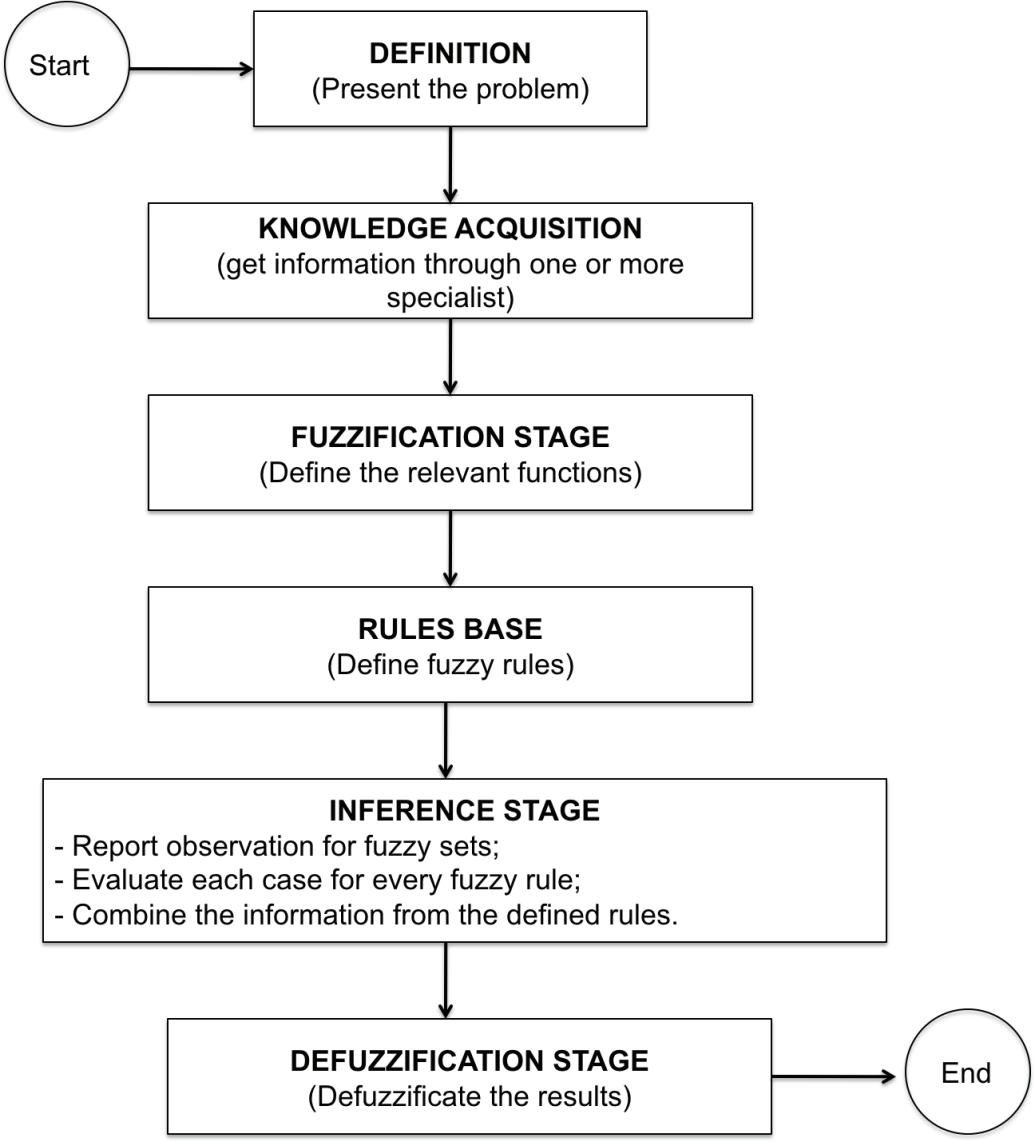
**Flowchart of creation and use of the fuzzy medical system**.

In complex systems and processes, are required mechanisms for dealing with inaccurate information and reasoning and processing procedures to make them tractable. An effective strategy in these circumstances involves the acquisition, representation and processing of concepts described linguistically using fuzzy logic. In the literature studied and towards the proposed problem was found that the strategy of applying fuzzy logic could provide more benefits (acquisition of specialist's knowledge, generation of rules base, the process automation and increased accuracy of the pre-diagnosis) and satisfactory results. The implementation of intervention and control actions, in the model developed uses fuzzy logic, considering that it enables the capture of specialists' knowledge in the same way that lets you check the precise timing of the intervention and alarm. We developed a flowchart to assist the creation and use of a fuzzy system, called medical fuzzy system, shown in Figure 3.

The fuzzy medical system definition and its stages (Fuzzification Stage, Inference Stage, Defuzzification Stage and Rules Base) are presented below.

**1) Definition of fuzzy medical system:**conditions of vital signs analysis, considering the parameters of normality and the defined fuzzy rules base, inferences in vital signs are made generating alarms from pre-diagnosis indicating abnormalities.

**2) Obtain information from one or more specialists:**the role of a specialist in the application to be modelled is of fundamental importance to collaborate in the construction of membership (relevants) functions for the entries description.

**3) Define the fuzzy sets (membership functions) - Fuzzification Stage:**in this stage the input variables are defined identifying to which fuzzy set(s) they belong to by assigning the respective degree to each relevance. The fuzzy sets represented by relevance functions should be set on the discourse universe in order to understand it completely. Thus, before the creation of fuzzy system, it is necessary to assemble the fuzzy sets (relevance functions) to be used in both fuzzification and defuzzification stages. The inputs of fuzzy system in question are the main vital signs (mean blood pressure and partial oxygen saturation) that were defined by the following relevance functions.

**Figure 4:**
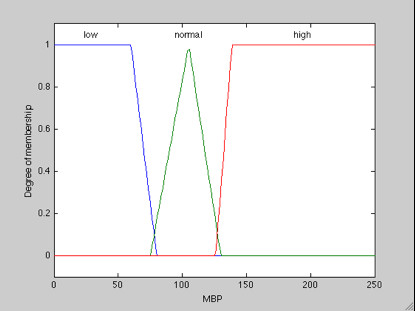
**Membership function of MBP**.

**a. Mean Blood Pressure (MBP) Membership**: MBP normal (N_MBP_) considering a domain (80-130), by the linguistic terms low (L), normal (N) and high (H), respectively representing the bands, as illustrated in Figure 4.

#### Fuzzy set of MAP

Low MBP (L_MBP_) < 80 → L_MBP_= {(0, 1), (60, 1), (80, 0)};

Normal MBP (N_MBP_) 80-130 → N_MPB _= {(75, 0), (105, 1), (130, 0)};

High MBP (H_MBP_) > 130 → A_MPB _= {(126, 0), (138.7, 1), (200, 1)}.

**Figure 5:**
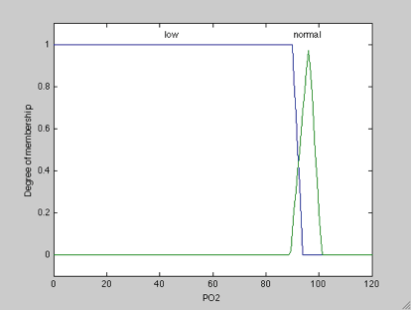
**Membership Function of POS_2_**.

**b. Partial Oxygen Saturation Membership POS**_2_: POS_2 _normal (N_POS2_) considering a domain (94-100), by linguistic terms, low (L) and normal (N), respectively, representing the bands, as illustrated in Figure 5.

#### Fuzzy set of POS_2_

POS_2 _baixa (B_POS2_) < 94 → B_POS2 _= {(0, 1), (90, 1), (94, 0)};

POS_2 _normal (N_POS2_) 94 - 100 → N_POS2 = _{(89.2, 0), (96.2, 1), (100,0)}.

For this purpose, relevance functions were built from the direct method, the specialist informed all relevance functions data (values that represent each function and the degree of relevance, within the function of each) in order to set them explicitly. It is noteworthy that there are many relevance functions, but the most used in this fuzzy system were the triangular and trapezoidal as they better represent the functions according to the context.

**4) Fuzzy rules definition (Rules Base):**the rules base is assembled with the following structure: If < premises > Then < conclusion >. For the rules definition of the fuzzy medical system concerned, we could standardize the following structure:

• R: {R_1_, R_2_, R_3_,...,R_n_} → Set of rules;

• SV: {SV_1_, SV_2_, SV_3_,..., SV_n_,} → Set of vital signs;

• D: {D_1_, D_2_, D_3_,..., D_n_} → Set of possible diagnoses;

• P: {n, l, h}→ Parameterization of signals (normal, low and high).

#### # Definição das Regras

#Rule: IF <(SV_1_, SV_2_, SV_3_,..., SV_n_,)> <{n(↓↑), a (↑), b (↓)}> And/Or <(SV_1_, SV_2_, SV_3_,..., SV_n_,)> <{n(↓↑), a (↑), b (↓)}>

THEN 

To exemplify, some rules for fuzzy medical system were set up, using two vital signs < premises > and 6 (six) situations as pre-diagnosis.

**# Rule 1**: If low MAP and low POS2 then there is clinical instability.

IF MBP ↓ AND POS2↓ THEN UNSTABLE

**# Rule 2: **If low MBP and normal SPO2 then low MBP.

IF MBP ↓ AND POS2↓ ↑ THEN ↓ MBP

**# Rule 3: **If normal MBP and low SPO2 then hypoxemia.

IF MBP ↓ ↑ AND POS2↓ THEN HYPOXEMIA

**# Rule 4: **If normal MBP and normal SPO2 then stability in clinical condition.

IF MBP ↓ ↑ AND POS2↓ ↑ THEN STABLE

**Rule # 5**: If high MBP and low SPO2 then instability in the clinical condition.

IF MBP ↑ AND POS2↓ THEN UNSTABLE

**# Rule 6: **If high MBP and normal POS2 then high MBP

IF MBP ↑AND POS2 ↓ ↑ THEN ↑MBP

At this stage it is important that the amount of rules defined can cover all possible combinations of inputs and outputs of the problem proposed and that the consistency of the rules is reviewed to avoid contradictions. The rules base was developed from several meetings, discussions and interviews with the Promater hospital medical staff.

**Figure 6:**
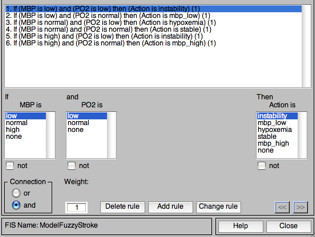
**Rules Base**.

**5) Reporting the comments to the fuzzy sets - Inference Stage: **At this stage, the inputs are analyzed to generate the output fuzzy set with its respective degree of compatibility. In the proposed fuzzy medical system, we used the controller model proposed by Mamdani [25], where the activation function of each rule is set in and the inference system determines the degree of compatibility with the premise of the rules contained in the rules base. After that, it is determined which rules were activated and the relevance output function is applied, joining all activated output fuzzy sets and their degrees of compatibility in a single Output Set (OS). This OS represents all actions that are acceptable to the input set, each one with their level of compatibility. It is also assessed at this stage, each case for all fuzzy rules and the information combination is performed from the rules already defined in the Rules Base, as illustrated in Figure 6.

**6) Defuzzificate results - Defuzzification stage: **this stage is used to generate a single numerical value, from all possible values contained in the fuzzy set obtained from the inference stage, to generate the control action. As a consequent action of the relations and variability of vital signs, was adopted for defuzzification the field (0-10), as illustrated in Table 2.

**Table 2:** Diagnostics and levels of urgency of clinical cases

Cases	MBP	SPO2	Situation	Message	What should be done	Level of urgency
**1**	Low	Low	Unstable clinical situation (Instability)	The patient's vital signs are altered	Send alert to the doctor	High priority
**2**	Low	Normal	Low MBP	The patient's blood pressure is low		Low priority
**3**	Normal	Low	Hypoxemia	Patient with hypoxemia - abnormal deficiency of oxygen concentration in arterial blood		High priority
**4**	Normal	Normal	Normal clinical situation (stable)	No alert	No abnormality	
**5**	High	Low	Unstable clinical situation	The patient's vital signs are altered	Send alert to the doctor	Medium priority
**6**	High	Normal	High MBP	The patient's blood pressure is high		Low priority

**a. Action Relevance Function (A) - Defuzzification: **representing the bands [< 2.5; 2.5-4.5; 4-6, 5, 5-8 and> 8] by linguistically terms instability, low MBP, hypoxemia, stable and high MBP.

### Alert Generation and Transmission

From the level of urgency set in the rules base already defined, messages are sent to the medical team from the issuance of alarms for devices (mobile or not) and may, according to pre-defined settings, be sent to desktop screen, via email, SMS and others. The post-processing and alerts sending is the architecture mechanism responsible for the control, sending and receiving messages between users and the architecture. This way, it is possible to create an effective communication system between a user (doctor) and the station (ICU). The main idea is that medical staff can connect to the centre with wireless network coverage in the hospital and thus receive on their device the alerts of possible changes in vital signs of monitored patients, as defined by Araujo [26].

### Validation

The simulations and validations of the proposed architecture have been conducted using MATLAB 2009a (student version) because of the tools available in this application for the development of models and the rapid visualization of the results obtained in the fuzzy system.

The fuzzy model developed for pre-diagnosis of patients in the ICU performs the interaction between the captured values, operated by the inference rules in fuzzy expert system, triggering control actions, monitoring and helping to medical diagnosis. The model indicates the alarms in accordance with the Guideline (Jauch et al., 2010), prescribed and developed by the American Heart Association, playing the main features defined for issuing alerts. After obtaining the definition of normal values of vital signs, the relevance functions of the main variables that directly influence the clinical condition of ICU patients were acquired. For the generation of rules base was asked to physicians, considering the parameters of normality and abnormality, to indicate the diagnosis of each clinical case and their level of urgency as already illustrated in Table 2.

To validate the SIMAp the patient's condition was monitored and classified in five situations that can significantly alter the clinical condition of ICU patients: 1-clinical instability (all signs altered), 2- low MBP; 3-hypoxemia; 4-stable, and 5- high-MBP.

We adopted the basis of fuzzy rules taking as background a normal MBP, considering a domain [80, 130], representing the bands [< 80, 80-130] and [> 130] by linguistic terms low, normal and high, respectively; and partial oxygen saturation, considering a domain [94,100], representing the bands [< 94 and 94-100] by linguistic terms low and normal, respectively.

As the consequent action of signs relations and variability, it was adopted for defuzzification the domain [0,10], representing the bands [< 2.5; 2.5 - 4.5; 4-6; 5.5 - 8 and> 8] by linguistic terms: instability, low MBP, hypoxemia, stable, high MBP, respectively.

Therefore, we used the vital signs selections required for the entry of fuzzy model constructing inference by relevance functions, rules base and already defined alarm conditions of vital signs for monitoring and support medical diagnosis in ICU inpatients.

**Figure 7:**
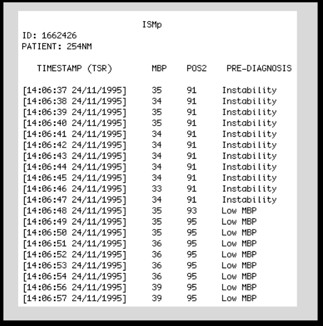
**Diagnosis of Register 254N**.

**Figure 8:**
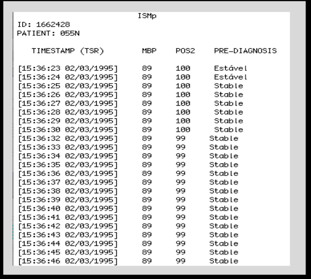
**Monitoring and diagnosis of Register 055N**.

An important step in this process was the extraction of data relevant to the model functioning, since the record is composed of many signs that are not used, which could interfere with the results and performance of the model. In the processing and sorting stage the results of the clinical diagnosis of patients' records were obtained through the model, as illustrated in Figure 7 and 8

In Figure 7, we obtained the monitoring extract (query) of the patient identified by ID: 1662426 - 254 NM, from 14 h: 06 m: 37 s of the day 24/11/1995 to 14 h: 06 m: 57 s of the day 24/11/1995. It was noted that monitoring is conducted every second and that in this interval the patient started unstable and passed to the state of Low MBP.

In Figure 8, we obtained the monitoring extract (query) of the patient identified by ID: 1662428 - 055 NM, from 15 h: 36 m: 23 s of the day 02/03/1995 to 15 h: 36 m: 46 s of the day 24th/11/1995. The monitoring was also performed every second and in the interval the patient presented a stable situation.

## Results

In the implementation of the model for monitoring and supporting the medical diagnosis of the clinical situation of the ICU patients was obtained a satisfactory result, with 96% accuracy (including the five situations planned) and 4% false alarms (due to various causes: from calibration of the equipment itself to body movements), according to analysis conducted by medical experts through the inferences made.

To this outcome measurements were available 100 (one hundred) inferences through forms (with patients' vital signs) so that physicians involved in this project could validate the pre-diagnosis provided by the proposed architecture and the fuzzy model.

It should be noted that medical Specialists are part of the Promater Hospital who promptly answered the questionnaire separately and later joined together to discuss the results, they are both critical care physicians.

**Figure 9:**
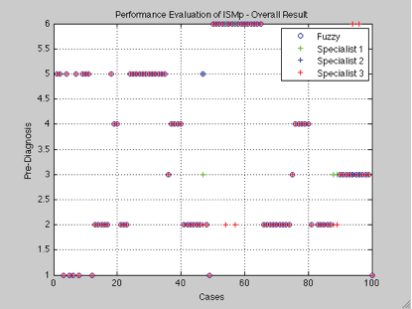
**Performance Evaluation of ISMp - Overall Result**.

Analyses were performed by the answers provided by medical forms and discussions of results. In Figure 9 you can view the overall performance evaluation of the model through the fuzzy output and results of three specialists. It was obtained from 100 hundred inferences: 92 (ninety-two) with similar diagnoses and 8 (eight) with different diagnoses. After discussing the results with the specialists it was verified that between the 8 (eight) with different diagnoses: 4 (four) the fuzzy inferred correctly, and 4 (four) diverged from each other, as illustrated in Table 3.

**Table 3:** Divergent Cases

Divergent Cases	MBP	POS2	Fuzzy	SP 1	SP 2	SP 3	Discussion
**1**	33	94	Low MBP	Instability	Low MBP	Instability	Low MBP
**2**	126	87	Hypoxemia	Hypoxemia	Instability	Instability	Instability
**3**	123	87	Hypoxemia	Hypoxemia	Instability	Instability	Hypoxemia
**4**	126	87	Hypoxemia	Hypoxemia	Instability	Instability	Instability
**5**	36	92	Low MBP	Instability	Instability	Instability	Instability
**6**	40	92	Low MBP	Instability	Instability	Instability	Instability
**7**	117	94	Stable	Hypoxemia	Instability	Hypoxemia	Stable
**8**	98	94	Stable	Hypoxemia	Stable	Hypoxemia	Stable

The use of ANNs and fuzzy logic in medical applications can be very useful if considered as an auxiliary tool for health professionals. Thus, applying the results already achieved and considering that so far there is no record of the diagnosis resulting from the co-relation between the vital signs analyzed, inferences derived from the fuzzy model were applied in the training and validation of an ANN to classify results, as described below.

**Figure 10:**
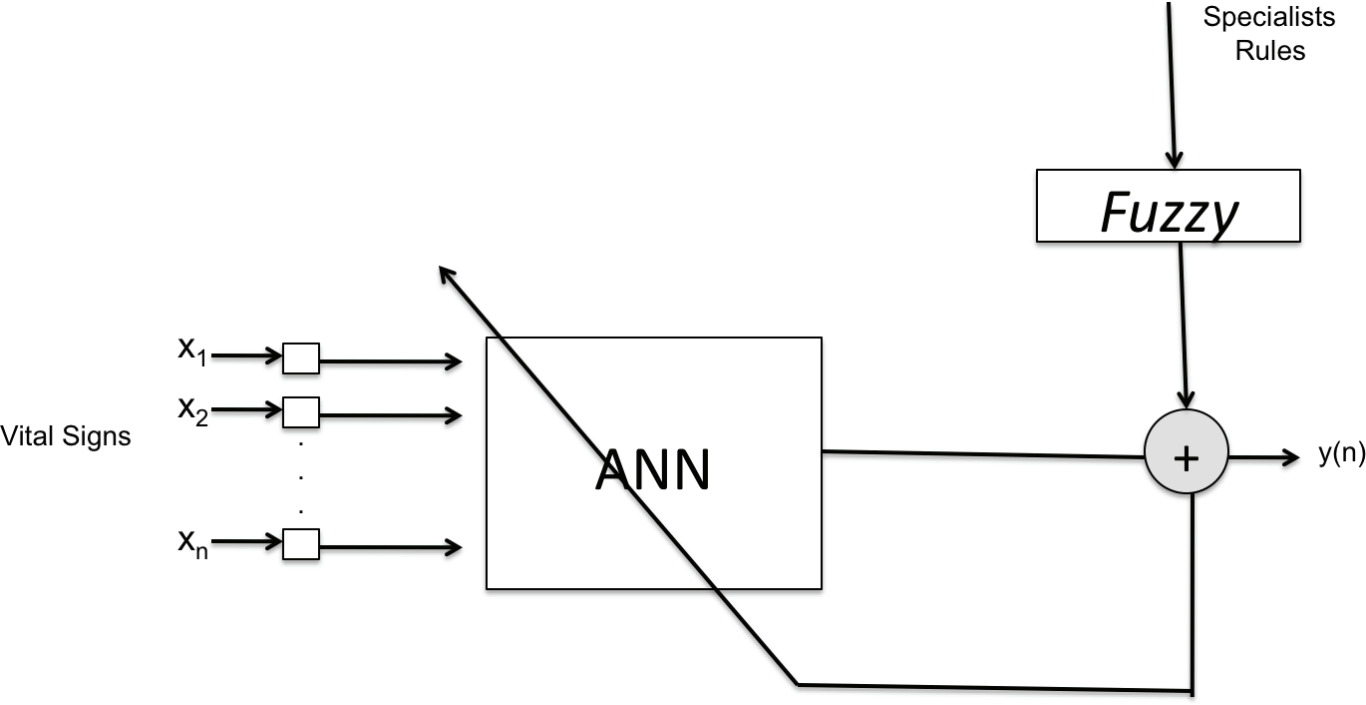
**Integration of ANN with Fuzzy**.

It was found that integration of ANN with the inferences (pre-diagnosis) could provide interesting results for the classification of five cases (1-instability, 2- low MBP, 3-Hypoxemia; 4-Stable, and 5- high-MBP) predicted by the model, because until then the pre-diagnosis resulting from the junction of vital signs was unknown, as illustrated in Figure 10.

**Figure 11:**
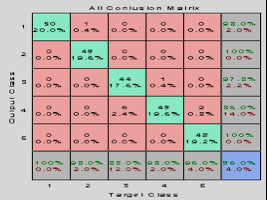
**Confusion Matrix**.

For this purpose it was necessary to mount a single database with all records to separate the training set and validation. The ANN architecture used in this case study was the MLP, with hyperbolic sigmoid tangent transfer functions, training Rprop (Resilient backpropagation) function and learning function of descent gradient, through a supervised learning. Regarding topology, neural networks with an inner layer were tested, and neurons ranged from 5 to 65. The satisfactory result was obtained with 15 neurons in the hidden layer. For the proposed problem, the implementation of ANN for the classification of the five proposed situations had a satisfactory result according to the confusion matrix generated, shown in Figure 11. The MLP neural network with backpropagation met the classification expectations and, thus, the objective of the work was contemplated with regard to aid the doctor in monitoring ICU patient's vital signs.

## Conclusions

This paper presented a fuzzy model able to monitor and classify the condition of the vital signs of hospitalized patients, sending information to an alerts system [25] according to the pre-diagnosis done helping the medical diagnosis.

Monitoring, processing, validation and testing of the fuzzy model were carried out using database with real data. It was also presented a simulation in order to evaluate its effectiveness, taking into account the pre-adjustment of the relevance functions in the pursuit of reducing false alarms. The use of fuzzy logic proved be for to the medical area very useful as a tool to assist specialists in this area.

Finally, from this study numerous possibilities for future work arise, such as: validation of the model in a real scenario and environment, confronting the real-time alarm generation and reception of messages being evaluated by the patient's physician, an increase of vital signs in the model, inclusion of specific alarms for each patient.

## Competing interests

The authors declare that they have no competing interests.

## Authors' contributions

The main contributions are: CL participated of the mapping of the vital signs that influence the clinical status of ICU patients, conducted from contact with specialists (doctors); GS conceived of the proper parameterization of the vital signs relevance functions; RV help of the definition of the rule base including the margin of normality in each vital signs parameter and the parameters setting for the correct model defuzzification applied to ICU patients; AN conceived of the parameters adjustment to fit the correct defuzzification of the model applied to ICU patients, and integration of the fuzzy model and ANN to the classification of the patients' clinical situation and a pre-diagnosis system for medical staff. CL and AG conceived of the study, and participated in its design and coordination; All authors read and approved the final manuscript.
